# Dietary supplementation of squalene increases the growth performance of early-weaned piglets by improving gut microbiota, intestinal barrier, and blood antioxidant capacity

**DOI:** 10.3389/fvets.2022.995548

**Published:** 2022-11-03

**Authors:** Yang Gao, Xue Ma, Yingqing Zhou, Yongqiang Li, Dong Xiang

**Affiliations:** ^1^College of Life Science, Baicheng Normal University, Baicheng, China; ^2^College of Animal Science and Technology, Jilin Agricultural University, Changchun, China; ^3^Muyuan Joint Stock Company, Nanyang, China

**Keywords:** squalene, growth performance, gut microbiota, intestinal barrier, early-weaned piglets, blood antioxidant capacity

## Abstract

This study aimed to investigate the effects of dietary squalene (SQ) supplementation on the growth performance of early-weaned piglets. Twenty early-weaned piglets were randomly divided into two groups, the squalene group (SQ) and the control group (CON). The CON group was fed a basal diet, and the SQ group was fed a basal diet with 250 mg/kg squalene. The feeding period lasted 21 days. The results showed that SQ significantly increased the final body weight (FWB, *P* < 0.05), average daily gain (ADG, *P* < 0.05), and average daily feed intake (ADFI, *P* < 0.05) and significantly decreased the F/G ratio (feed intake/gain, *P* < 0.05) and diarrhea index (DI, *P* < 0.05). In terms of blood biochemical indicators, SQ significantly increased anti-inflammatory factors such as transforming growth factor-β (TGF-β, *P* < 0.001), interleukin-10 (IL-10, *P* < 0.001), and interferon-γ (IFN-γ, *P* < 0.01), and decreased pro-inflammatory factors such as tumor necrosis factor-α (TFN-α, *P* < 0.001) and interleukin-6 (IL-6, *P* < 0.001). Furthermore, SQ significantly increased blood antioxidant indexes (*P* < 0.001) such as superoxide dismutase (SOD), glutathione peroxidase (GSH-Px), catalase (CAT), and total antioxidant capacity (T-AOC) and significantly decreased the level of malondialdehyde (MDA) (*P* < 0.001). The villus height (*P* < 0.001) and V/C ratio (villus height/crypt depth, *P* < 0.001) of the jejunum were significantly increased in the SQ group, while the crypt depth (*P* < 0.01) was decreased compared to the CON group. The intestinal permeability indexes, namely diamine oxidase (DAO), D-lactic acid (D-Lac), regenerative insulin-derived protein 3 (REG-3), and FITC-Dextran 4 (FD_4_), significantly decreased the concentrations in the treatment group (*P* < 0.001), and the antioxidant indexes of the jejunum, such as SOD, GSH-Px, CAT, and MDA, were improved by adding SQ. The qPCR results showed that adding SQ could significantly increase the mRNA expression of jejunal tight-junction proteins, such as zonula occludens-1 (*ZO-1, P* < 0.001), *Occludin* (*P* < 0.001), *Claudin* (*P* < 0.001), glucagon-like peptide-2 (*GLP-2, P* < 0.001), and insulin-like growth factor-1 (*IGF-1, P* < 0.001). Then, we used Western blotting experiments to further confirm the qPCR results. In addition, it was found that adding SQ increased the abundance of beneficial bacteria such as *Gemmiger* (*P* < 0.01) and decreased the abundance of harmful bacteria such as *Alloprevotella* (*P* < 0.05), *Desulfovibrio* (*P* < 0.05), and *Barnesiella* (*P* < 0.05). It was interesting that there was a very close correlation among the fecal microbes, growth performance parameters, intestinal barrier, and blood biochemical indicators. In conclusion, the data suggest that SQ supplementation could effectively improve the growth performance of early-weaned piglets by improving the gut microbiota, intestinal barrier, and antioxidant capacity of the blood and jejunal mucosa.

## Introduction

After early weaning, piglets often experience weaning stress, which manifests as diarrhea and reduced performance due to gut barrier dysfunction (1, 2). However, the most effective way to solve such problems is to use antibiotics (3), because they can not only improve the growth rate (4) and feed utilization (5) and reduce mortality (6) in livestock husbandry but also kill harmful bacteria in the intestine, reducing the occurrence of diarrhea and intestinal inflammation (7). In recent years, following the European Union, China has completely banned the use of antibiotics in swine feeds, so more and more green feed additives, especially natural plant extracts, have received extensive attention from animal nutrition experts.

Squalene (SQ) is a triterpenoid unsaturated hydrocarbon that plays a significant role in the synthesis of fat-soluble vitamins, hormones, and cholesterol in eukaryotes (8). It contributes greatly to immune regulation, antioxidation, anti-inflammatory response, anti-cancerous responses, defense against degenerative diseases, and improvement of cardiovascular health (9, 10). The research by Clara et al. (11) showed that supplementing the diet with SQ could increase the amount of high-density lipoprotein (HDL) and paraoxonase-1 in the serum of mice and alleviate oxidative stress damage (11). SQ also had a crucial effect on alleviating isoproterenol-induced oxidative stress in the hearts of rats (12). In addition, SQ can increase serum glutathione (GSH), superoxide dismutase (SOD), and catalase (CAT) levels in myocardial infarction model rats. In recent years, the research on SQ has been mostly focused on production technology, material development, separation, purification, and identification. However, there are very few studies on the growth performance and intestinal barrier in livestock production. Therefore, it is necessary to study these factors for the development of SQ in the livestock breeding industry, especially in terms of the production performance of animals.

As is well-known, the microflora in the gastrointestinal tract plays an important role in animals. In the digestive tract, the jejunum has received extensive attention because it is closely related to digestion and absorption (13). It has been demonstrated that the gut microbial composition of pigs can affect the intestinal barrier. On the one hand, increased harmful bacteria can disrupt the intestinal barrier, resulting in an inflammatory response and reduced growth performance (14). On the other hand, increased levels of beneficial bacteria such as butyrate-producing bacteria can repair the gut barrier (15). Therefore, the composition of the gut microbiome is closely related to the gut barrier. However, current research has shown that SQ can improve the growth performance of broilers (15), but its effect on the intestinal barrier and microorganisms is unknown. There is even less research in swine fields. Therefore, the current study aimed to investigate the potential mechanism of dietary squalene supplementation on growth performance, intestinal barrier, and gut microbiota in early-weaned piglets, and the results would provide guidance and reference for its further application as a promising feed additive in swine feed.

## Materials and methods

All animal procedures were approved by the Animal Care and Use Committee of Baicheng Normal University, and experiments were approved by the Committee of Experimental Animal Welfare and Ethical of Baicheng Normal University (BNU2020-14).

### Animal care and experimental design

**Figure 1:**
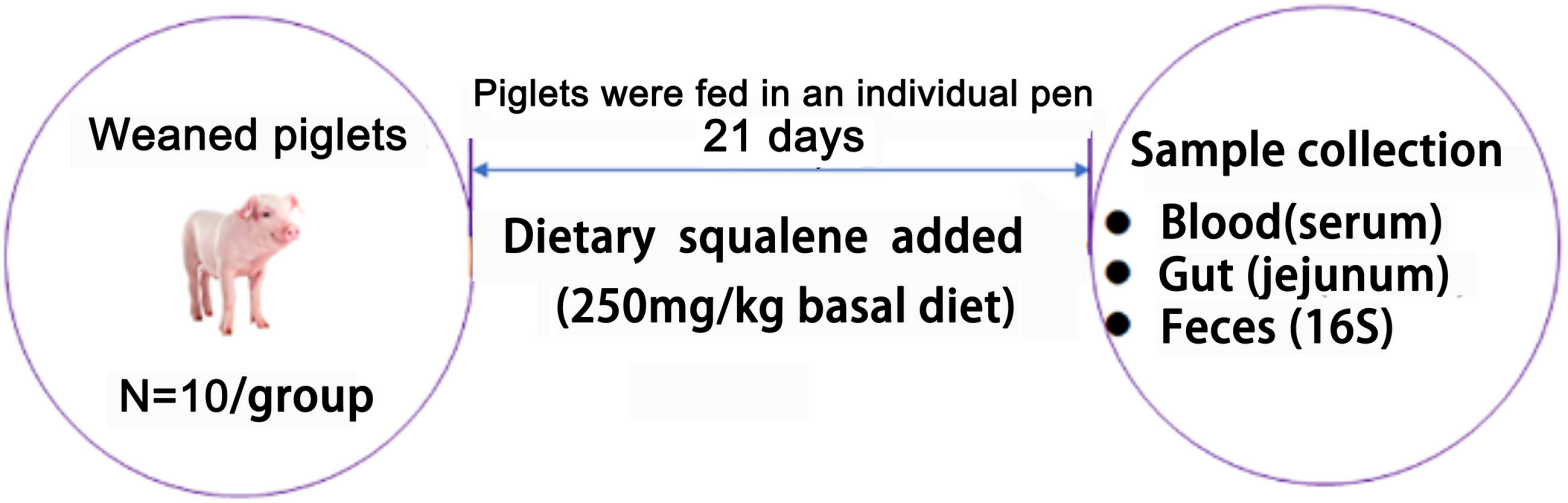
Study scheme.

Twenty early-weaned piglets (three-way-crossed) were selected and randomly divided into two groups, the squalene group (SQ) and the control group (CON). The CON group was fed a basal diet, whose nutritional composition is shown in Supplementary Table 1, and the SQ group was fed a basal diet with 250 mg/kg (16) squalene, which came from Dahaigui Life Science Co., Ltd., with a purity of ~92%. The feeding period lasted 21 days (17). The experimental design is shown in Figure 1. Each piglet was raised in an individual pen irradiated with natural light, and the ambient temperature was kept at 24–25°C. The swine fever vaccine was injected when piglets were 28 days old, and the pseudorabies vaccine was injected when they were 35 days old. The initial body weight was recorded before the experiment, the final body weight was recorded after the experiment, and the feed intake was recorded every day. The average daily gain (ADG) = (final weight–initial weight) divided by the number of days. The average daily feed intake (ADFI) = total feed intake divided by the number of experimental days. The diarrhea index (DI) evaluation method was referenced (18). During the experimental period, the health and diarrhea statuses were observed and recorded every day. Scoring was performed according to fecal morphology, and the score was divided into four grades, 0 = normal, 1 = mushy, 2 = semi-liquid, and 3 = liquid, where a score ≥2 was considered diarrhea. Diarrhea index = (sum of fecal scores)/(number of piglets × total number of days in the experiment). The breeding experiment was carried out in a farmer breeding cooperative in Lishu City, Jilin Province, in September 2020.

### Sample collection

At the end of the experiment, blood samples were collected and then centrifuged at 3,000 rpm for 10 min. The supernatant was taken, and the serum was stored at −80°C for analyzing. The jejunum tissues were taken out as soon as possible. One part was stored in 4% paraformaldehyde for histopathology examinations. The other parts were frozen in liquid nitrogen for further analysis. Fecal samples were taken from the rectum, then placed in liquid nitrogen, and finally stored in a −80°C freezer for 16S analysis.

### Hematoxylin and eosin staining and histopathological examination

Jejunum tissue was immersed and fixed with Carnoy's solution for 24 h. Then, the samples were removed from formalin and embedded in the paraffin. Subsequently, the paraffin blocks were sectioned to obtain 5 μm thick sections using a semi-automatic microtome (LONGSHOU, China). Sections were stained with hematoxylin and eosin (H&E) staining and viewed under an optical microscope. Histopathological examination was evaluated based on the villi height, crypt depth, and V/C ratio (18).

### Inflammatory cytokines and antioxidant analysis

The levels of pro-inflammatory cytokines (TNF-α, IL-6), anti-inflammatory cytokine (IL-10, TGF-β, IFN-γ), and antioxidant-related parameters (T-AOC, SOD, GSH-Px, CAT, and MDA) in serum were tested using ELISA kits (Nanjing Jiancheng Bioengineering Institute, Nanjing, China) following the instructions of the manufacturer. Meanwhile, antioxidant-related parameters (T-AOC, SOD, GSH-Px, CAT, and MDA) in jejunum tissues were measured using the same methods.

### Intestinal permeability assay

The Ussing Chamber Technology was used following previous studies (19, 20); here, we used an Ussing Chamber with a 1.13 cm^2^ hole to mount tissues that could be washed with a Ringer solution. After we absorbed the agarose solution till it was viscous and translucent, we absorbed the agarose solution in a syringe, inserted the electrode tip into the syringe, and observed that there were no air bubbles in it. The electrode tip was successfully prepared. Then, we assembled the chamber system, corrected the electrodes, and stripped off the serosal membrane of the intestine before installing it on the Ussing chamber. We then fixed the intestinal mucosa sample in the system, adjusted the current and voltage, opened the chamber system, and connected the Acquire & Analyze software on the computer. After the equilibration period (6 h), the transepithelial electrical resistance (TER) can be calculated, and the whole procedure lasted 30 min. For 1 h, TER (Ω cm^2^) is recorded every 15 min. In this condition, the average data of the TER values represent specific pigs. The mucosal permeability can be tested using the fluxes of fluorescein isothiocyanate-dextran 4 kDa (FD_4_). This method is suitable for most mammals, especially humans and pigs (21). We put the mucosa in the bathing reservoir in Ussing Chambers to place 4 mg/ml FITC-dextran on it. Next, the quantity of FD_4_ on the serosal side was measured every 30 min (for a total of 90 min) through a fluorescence plate reader at 492 nm. The wavelength of extinction was 520 nm. Mucosal injury markers diamine oxidase (DAO), D-Lactate (D-Lac), and regenerative islet-derived protein 3 (REG3) of the jejunal mucosa were tested using commercialization kits (Jiancheng Bioengineering).

### Quantitative real-time polymerase chain reaction assay

The qPCR assay was followed by the previous study (22). Total RNA from the jejunum samples was extracted using Trizol reagent (Invitrogen, United States), chloroform, isopropanol, and 75% ethanol solution and then treated with DNase I (TaKaRa, China) for possible DNA contamination. The concentration of each RNA sample was quantified using the NanoDrop 2000. The HiFiScript cDNA was generated using the Prime Script RT Master Mix (TaKaRa, China) according to the manufacturer's instructions. The reverse transcription was conducted at 37°C for 15 min and 85°C for 5 s. qPCR was conducted using the KAPA SYBR FAST qPCR Master Mix kit according to the manufacturer's instructions. In brief, 1 μl of cDNA template was added to a total volume of 10 μl containing 5 μl of KAPA SYBR FAST qPCR Master Mix Universal, 0.4 μl of PCR forward primer, 0.4 μl of PCR reverse primer, 0.2 μl of ROX low, and 3 μl of PCR-grade water (KAPA Biosystems, United States). All samples were run in an Applied Biosystems 7,500 RT-PCR System (Thermo Fisher Scientific, China). Relative gene expression was normalized to the housekeeping gene GADPH and calculated using the 2^−ΔΔCt^ method, where ΔC_*t*_ = C_t_ (Target)–C_t_ (GAPDH). Primer sequences were designed using Primer 6.0 software and synthesized by Sangon Biotech Co., Ltd. (Shanghai, China). The primers used in this study are listed in Supplementary Table 2.

### Western blotting assay

The Western blotting method was performed following previous studies (23, 24), and the expression of tight junction proteins Occludin, Claudin, ZO-1, GLP-2, IGF-1 was investigated. Total protein was extracted by using the CWBIO kit, Beijing, China. Pierce™ BCA Protein Assay Kit (Thermo Scientific, Rockford, USA) was used to determine jejunum protein, and the sample proteins had to be stored at −80°C for further analysis. The SDS-PAGE method was used to separate the protein and transfer it onto a polyvinylidene difluoride (PVDF) microporous membrane for 10 min by a semi-dry transfer film method. In this case, the membrane can be blocked by 3% bovine serum albumin (BSA) for 2 h at 17°C. After washing three times with 0.1% Tween-20 in TBS, the membranes were incubated with a primary antibody, which was diluted at 1:500 in TBST with 1% BSA overnight at 4°C. The next day, the membrane was incubated with goat anti-rabbit IgG and HRP-conjugated secondary antibodies for 2 h and thoroughly washed five times at 17°C. Finally, the membrane was washed again and incubated with Luminata Crescendo Western HRP substrate (Millipore, Bedford, MA, USA) for only 1 min in the dark and imaged by using the IS4000 Camera (Kodak, Beijing, China).

### Fecal microbiota analysis

The samples were analyzed by Shanghai OE Biotech. Co., Ltd. (Shanghai, China).

#### DNA extraction

Total genomic DNA of boar feces was isolated using an E.Z.N.A.^®^ Stool DNA Kit (Omega Bio-tek Inc., USA) following the manufacturer's instructions. DNA quantity and quality were analyzed using NanoDrop 2000 and 1% agarose gel.

#### Library preparation and sequencing

The V3–V4 region of the 16S rRNA gene was amplified using the primers 338F (5'- ACTCCTACGGGAGGCAGCAG-3') and 806R (5'-GGACTACHVGGGTWTCTAAT-3') with Barcode. The PCR reactions (total 30 uL) included 15 uL PhusionR High-Fidelity PCR Master Mix (New England Biolabs), 0.2 mM primers, and 10 ng DNA. The thermal cycle was carried out with an initial denaturation at 98°C, followed by 30 cycles of 98°C for 10 s, 50°C for 30 s, 72°C for 30 s, and a final extension at 72°C for 5 min. PCR products were purified using an AxyPrep DNA Gel Extraction Kit (Axygen Biosciences, USA). The sequencing libraries were constructed with NEB Next^®^ UltraTM DNA Library Prep Kit for Illumina (NEB, USA) following the manufacturer's instructions, and index codes were added. Then, the library was sequenced on the Illumina MiSeq 2500 platform (Illumina, USA), and 300 bp paired-end reads were generated at the Novo gene. The paired-end reads were merged using FLASH (V1.2.71). The quality of the tags was controlled in QIIME (V1.7.02); meanwhile, all chimeras were removed. The “Core Set” of the Greengenes database 3 was used for classification, and sequences with >97% similarity were assigned to the same operational taxonomic units (OTUs).

#### Analysis of sequencing data

OTU abundance information was normalized using a standard sequence number corresponding to the sample with the least sequences. The alpha diversity indices were calculated with QIIME (Version 1.7.0). PLS-DA was performed using R software (v2.15.3).

### Statistical analysis

Data are expressed as the mean ± SEM. *P* < 0.05 was considered a significant difference. Student's *t-*test (SPSS 21 software) was used to perform the statistical analyses. Spearman's correlation analysis was completed by RStudio (version 4.0.3) platform. Plots were performed by using GraphPad Prism 8.0.2.

## Results

### SQ improves the growth performance of early-weaned piglets

From Table 1, we can see that there was no difference in initial weight (*P* > 0.05) between the two groups. However, adding 250 mg/kg squalene to the basal diet significantly increased the final body weight (FBW, *P* < 0.05), average daily gain (ADG, *P* < 0.05), and average daily feed intake (ADFI, *P* < 0.05) of the early-weaned piglets compared to the control group. Meanwhile, SQ significantly reduced the F/G ratio (*P* < 0.05) and the diarrhea index (DI, *P* < 0.05). Therefore, dietary adding SQ can improve the growth performance of early-weaned piglets.

**Table 1:** The effects of squalene on the growth performance of weaned piglets.

**Items**	**Groups**	
	**CON**	**SQ**
Initial body weight, kg	5.33 ± 0.26	5.32 ± 0.23
Final body weight, kg	8.41 ± 0.17	8.77 ± 0.22*
Average daily gain, g/d	146.57 ± 13.65	169.28 ± 10.78*
Average daily feed intake, g/d	229.67 ± 15.45	247.15 ± 15.92*
F/G ratio	1.57 ± 0.08	1.46 ± 0.08*
DI	0.18 ± 0.06	0.08 ± 0.02*

There is no asterisk (^*^) in the same row means that the difference is not significant (P > 0.05), the asterisk (^*^) indicates the difference is significant (P <0.05).

### SQ improves anti-inflammatory factors and antioxidative stress in the serum of early-weaned piglets

**Figure 2:**
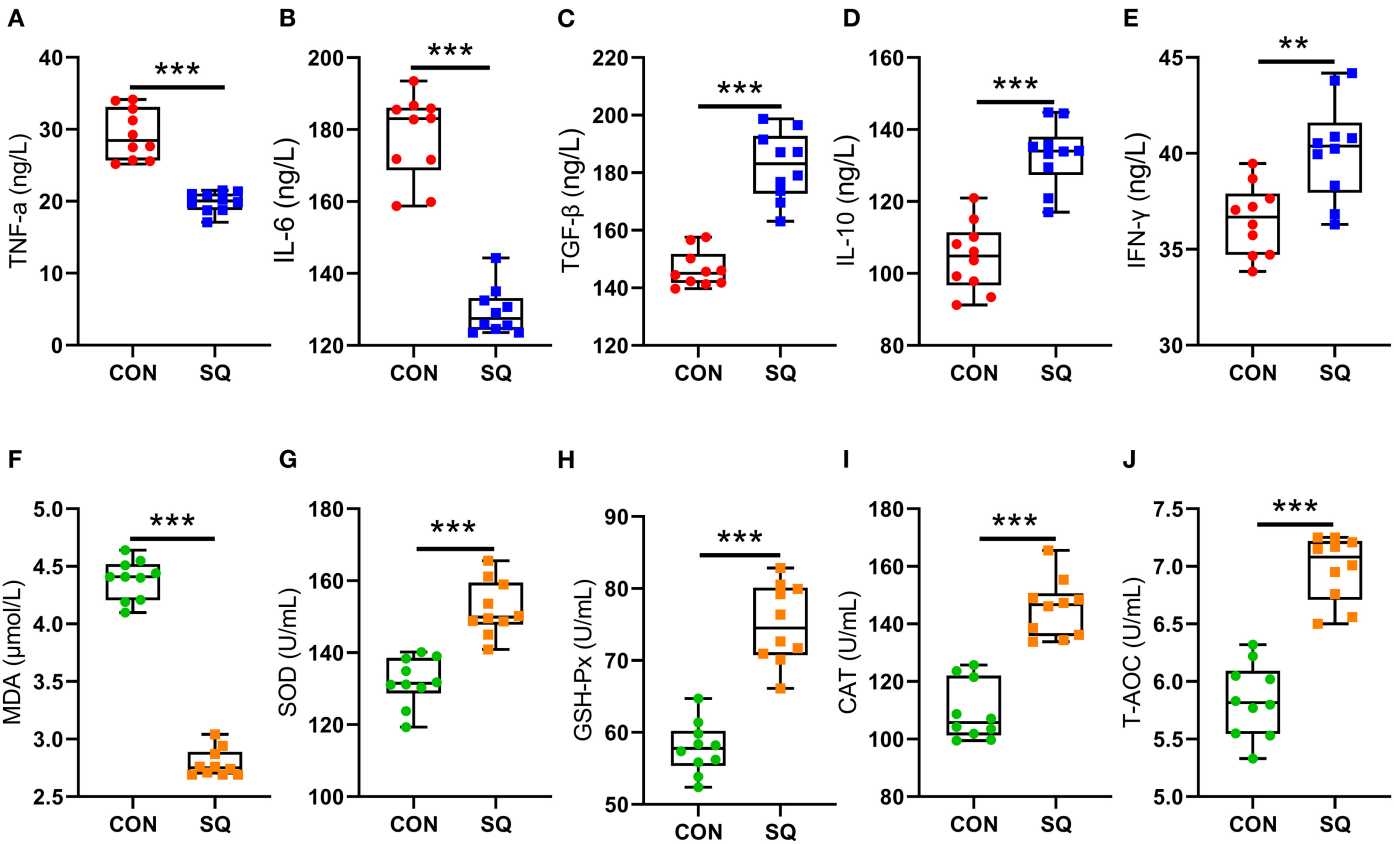
SQ changed blood biochemical indicators. **(A–E)** Pro-inflammatory factors and anti-inflammatory factors. Y-axis = amount, X-axis = the treatment groups. ** *P* < 0.01; ***P < 0.001. **(F–J)** Blood Antioxidant Indicators. Y-axis = amount, X-axis = the treatment groups. ***P < 0.001.

The results indicated that the SQ treatments reduced the levels of TNF-α and IL-6 significantly (Figures 2A,B, *P* < 0.001), while the SQ treatment dramatically increased the serum level of IL-10 (*P* < 0.001), TGF-β (*P* < 0.001), and IFN-γ (*P* < 0.01) compared to the CON group (Figures 2C–E). Furthermore, the serum level of T-AOC, GSH-Px, SOD, and CAT were significantly increased in the SQ group compared with the CON group (Figures 2G–J, *P* < 0.001). Meanwhile, the level of MDA in the serum was significantly decreased compared to the sCON group (Figure 2F, *P* < 0.001). Therefore, SQ can improve the anti-inflammatory and antioxidant capacity in early-weaned piglets.

### SQ benefits intestinal morphology and permeability in early-weaned piglets

**Figure 3:**
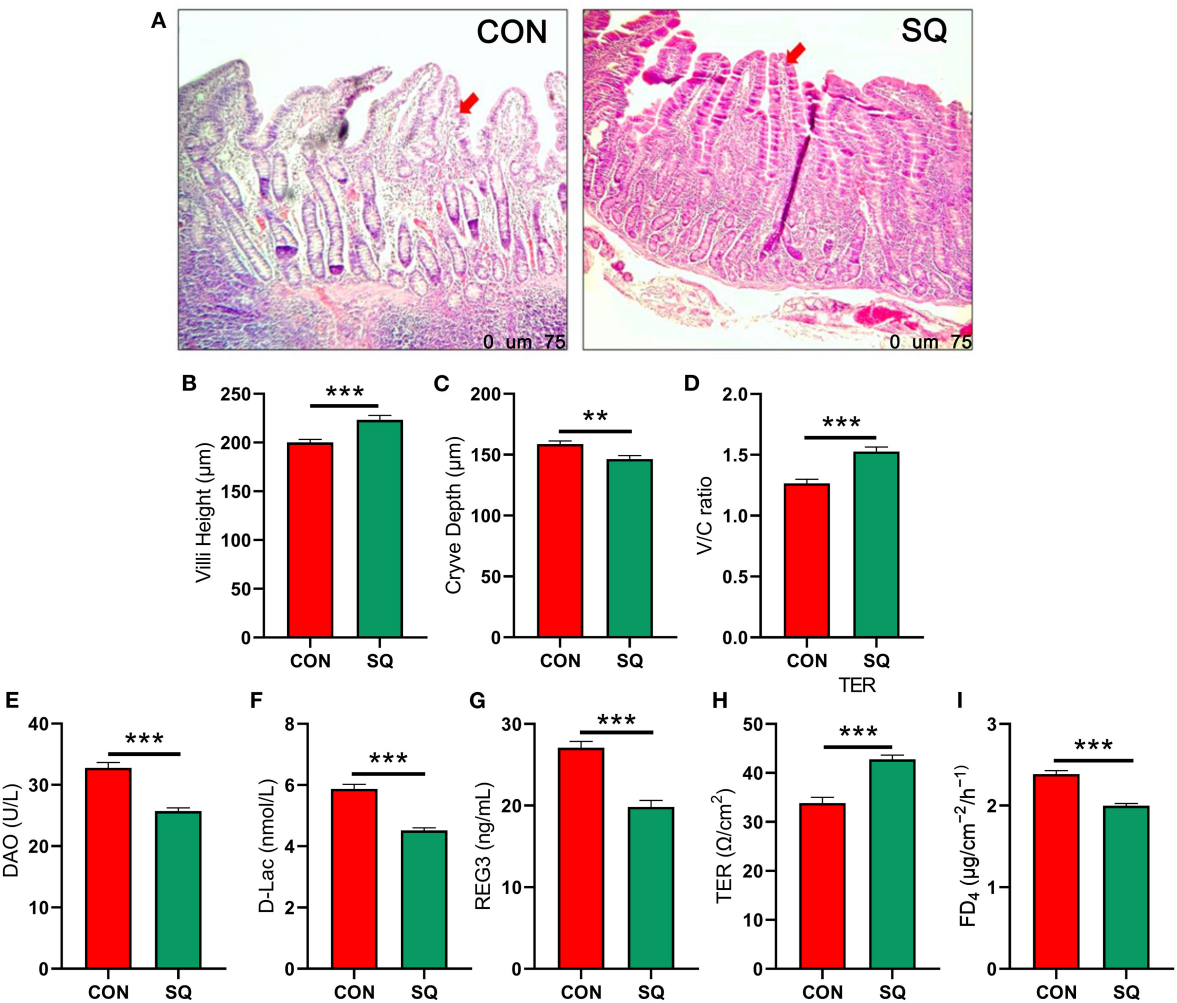
SQ improves intestinal permeability in early-weaned piglets. **(A)** H&E-stained sections of jejunum tissues between two groups under 40x microscope; **(B)** Jejunal villus height. Y-axis = length, X-axis = the treatment groups. ***P < 0.001. **(C)** Jejunal crypt depth. Y-axis = length, X-axis = the treatment groups. **P < 0.01. **(D)** Jejunal V/C ratio. Y-axis = relative abundance, X-axis = the treatment groups. ***P < 0.001. **(E–I)** Intestinal permeability indicators. Y-axis = amount, X-axis = the treatment groups. ***P < 0.001.

The intestinal morphology data are shown in Figure 3A. In the jejunum, dietary supplementation of SQ significantly increased villi height (Figure 3B, *P* < 0.001) and the V/C ratio (Figure 3D, *P* < 0.001) and decreased crypt depth (Figure 3C, *P* < 0.01). We also measured the indicators related to jejunum permeability, and the results showed that adding 250 mg/kg squalene to the basal diet significantly decreased the levels of DAO, D-Lac, and REG3 (Figures 3E–G, *P* < 0.001) in the jejunum mucosa. In terms of jejunal mucosal permeability, we tested the indexes TER and FD_4_, which were reflected in the gut barrier function. The results showed that squalene can significantly reduce the FD_4_ (Figure 3I, *P* < 0.001) and significantly increase the TER of the jejunal mucosa (Figure 3H, *P* < 0.001). Hence, the squalene benefits gut morphology and permeability in early-weaned piglets.

### SQ is beneficial for improving the intestinal barrier of early-weaned piglets

**Figure 4:**
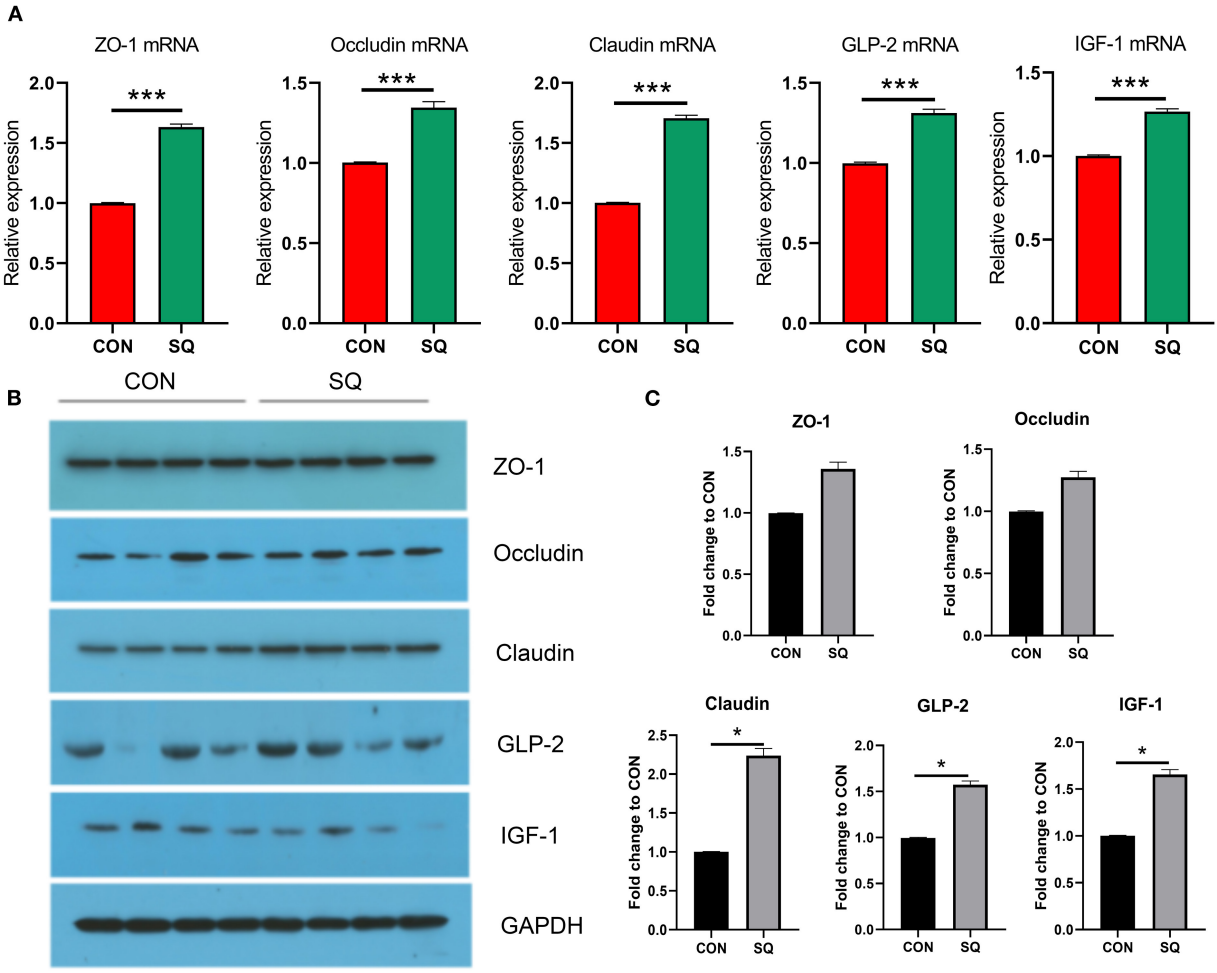
SQ is beneficial for improving the intestinal barrier of weaned piglets. **(A)** mRNA expression in the intestinal barrier. Y-axis = Relative expression, X-axis = the treatment groups. ***P < 0.001. **(B)** Tight junction protein levels of jejunum detected by Western blotting (N>3). **(C)** Quantitative data for western blotting analysis. Y-axis = fold change to CON, X-axis = the treatment groups (mg/kg body weight). * *P* < 0.05.

To further confirm the protective effects of SQ in the jejunal tissues, we used a combination of qPCR and Western blotting technologies to detect tight junction proteins associated with the intestinal barrier. The mRNA expressions of *ZO-1, Occludin*, and *Claudin* were significantly higher in the SQ group compared to the CON group (Figure 4A, *P* < 0.001). At the same time, we determined the mRNA expressions of *GLP-2* and *IGF-1*, which were associated with the growth in intestinal villi. The results indicated that the mRNA expressions of *GLP-2* and *IGF-1* in the SQ group were significantly higher than in the CON group (Figure 4A, *P* < 0.001). To further verify the results of qPCR, we used West blotting for verification. The WB results displayed in Figures 4B,C show that the protein levels of Claudin (*P* < 0.05), GLP-2 (*P* < 0.05), and IGF-1(*P* < 0.05) in the SQ group were significantly higher than those in the CON group. The expression levels of the other two proteins also showed an upward tendency in the treatment group. Therefore, squalene can improve the intestinal barrier function of early-weaned piglets by increasing the secretion of jejunal tight junction protein and promoting the growth of intestinal villi.

### Squalene improves intestinal antioxidant properties of early-weaned piglets

**Figure 5:**
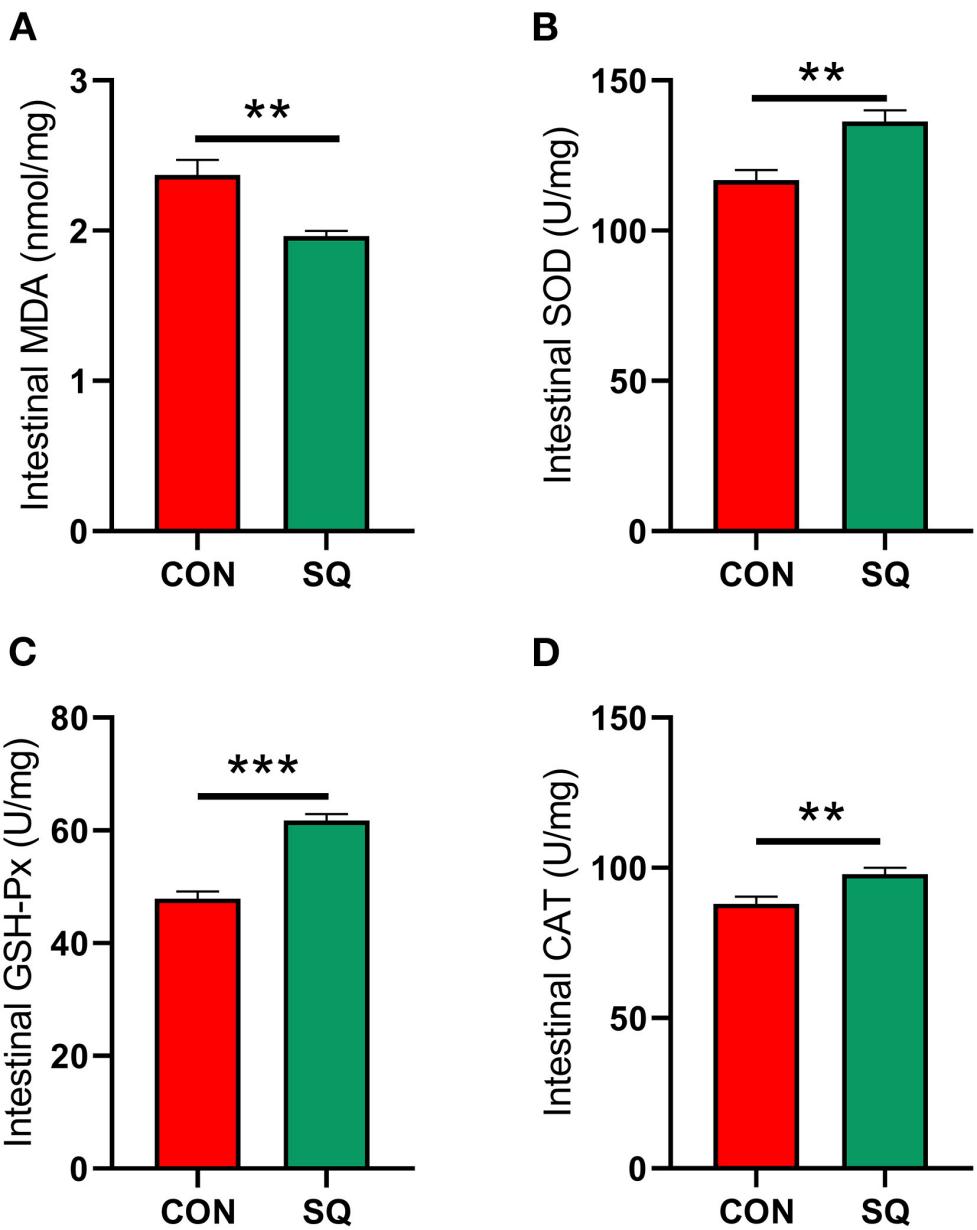
SQ benefits gut antioxidant capacity. **(A–D)** Antioxidant index of jejunum. Y-axis = amount, X-axis = the treatment groups. **P < 0.01; ***P < 0.001.

The results indicated that the antioxidant indexes such as SOD (*P* < 0.01), GSH-Px (*P* < 0.001), and CAT (*P* < 0.01) in the SQ group were higher than in the CON group (Figures 5B–D). In contrast, the level of MDA is lower in the treatment group (Figure 5A, *P* < 0.01). This means that adding squalene to the basal diet can improve the antioxidant performance of early-weaned piglets.

### Squalene is beneficial for improving gut microbial composition in early-weaned piglets

**Figure 6:**
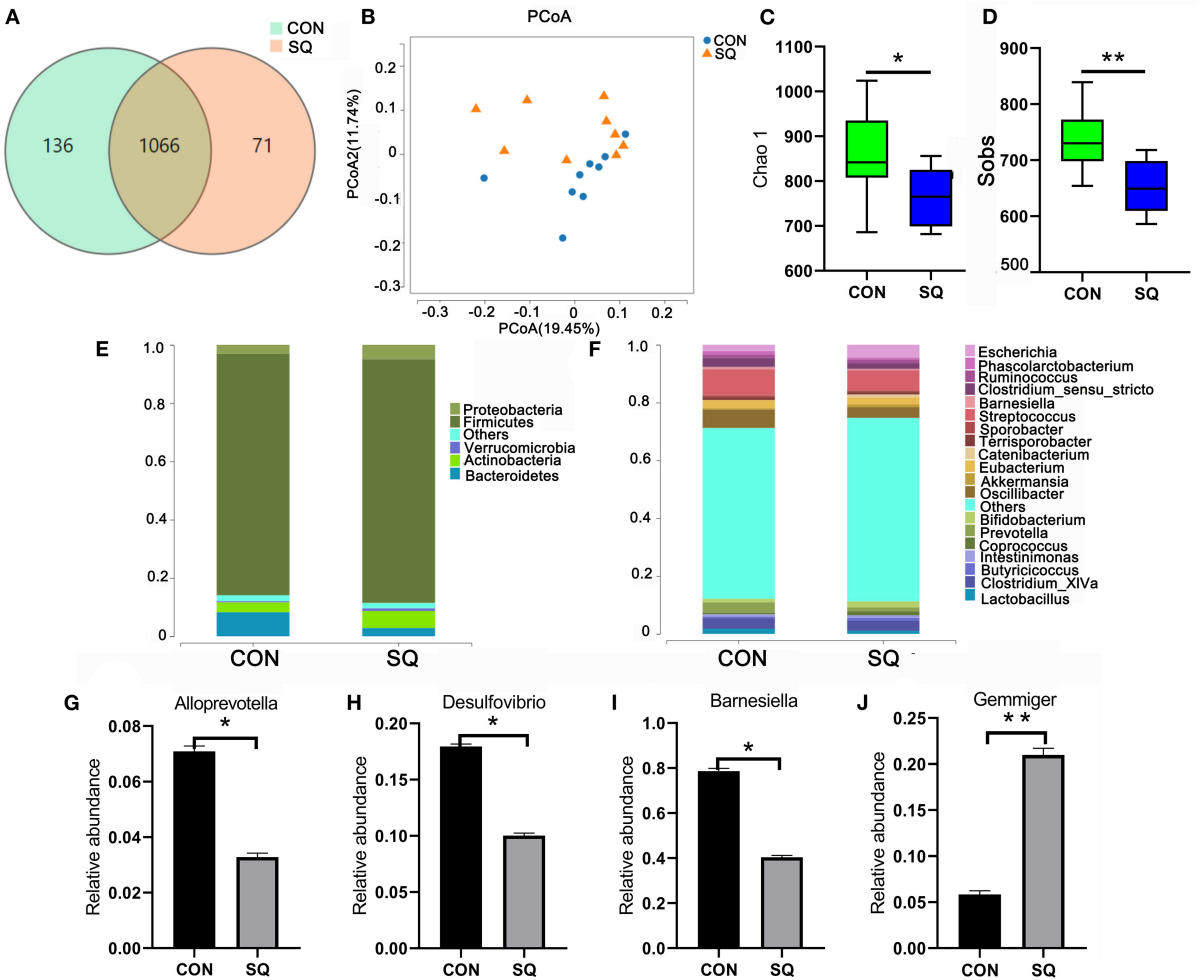
SQ improved gut microbiota of weaned piglets. **(A)** The Wayne figures of fecal microbiota. **(B)** PCoA analysis of fecal microbiota at the OUT level. **(C)** The Chao 1 index of fecal microbiota. **(D)** The Sobs index of fecal microbiota. **(E)** Relative abundance of fecal microbiota at the phylum level. **(F)** The relative abundance of fecal microbiota at the genus level. **(G–J)** Four altered microbes in genus level. Y-axis = relative abundance, X-axis = the treatment groups. **P < 0.01; *P < 0.05.

We used 16S rRNA sequencing to analyze the fecal microbiota. The Venn diagram shows that there were 1,066 common OTUs between the two groups and 71 and 136 unique OTUs in the SQ and CON groups, respectively (Figure 6A). β-diversity was conducted by principal coordinate analysis (PCoA) according to unweighted unifrac. The results showed that the gut microbiota in the SQ group was not significant compared to the CON group, but with a tendency (*P* = 0.08) (Figure 6B). α-diversity results showed that there were significant differences in the indexes of Chao 1 (*P* < 0.05) and Sobs (*P* < 0.01) between the two groups (Figures 6C,D). The microbial histograms at the phylum level and genus level between the two groups are shown in Figures 6E,F. At the genus level, we found that adding SQ to the basal diet significantly increased the prevalence of beneficial microbes such as *Gemmiger* (Figure 6J, *P* < 0.01) and decreased the prevalence of some harmful bacteria such as *Alloprevotella, Desulfovibrio*, and *Barnesiella* compared to the CON group (Figures 6G–I, *P* < 0.05). The microbes described above were all related to the growth performance of early-weaned piglets, so the SQ enhanced piglets' growth by changing microbial composition.

### Correlation analysis between growth performance, intestinal microbes, blood biochemical indexes, and the intestinal barrier

**Figure 7:**
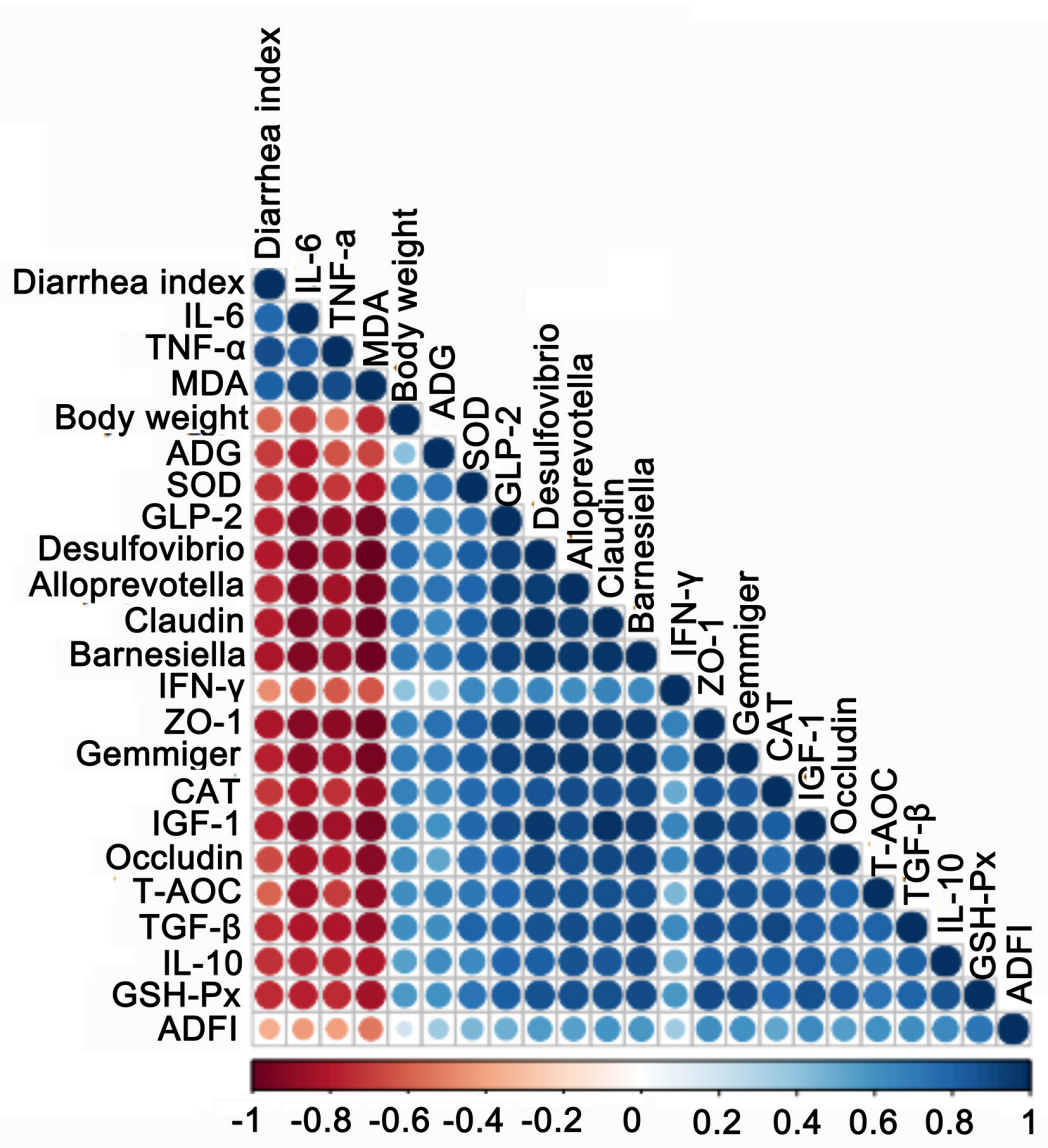
Correlation analysis among biochemical indexes, growth performance, gut barrier, and microbes. The color of the circle represents a positive or negative correlation, and the size of the circle represents the strength of the correlation (larger circle = stronger correlation).

To find the correlation between the growth performance, fecal microbes, and other biochemical parameters in early-weaned piglets by adding SQ, Spearman's correlation analysis was carried out based on experimental parameters. The correlation result is shown in Figure 7. We found that the growth performance of weaned piglets correlated well with gut microbiota, tight junction proteins, and antioxidant properties. The growth performance of body weight, ADG, and ADFI were all positively correlated with *Gemmiger*, ZO-1, and GLP-2; however, the DI was negatively correlated with all parameters except bad factors, such as MDA and IL-6. Therefore, SQ may improve the growth performance of early-weaned piglets by changing gut microbes, intestinal barrier, and blood antioxidant capacity.

## Discussion

In most cases, the weaning age of piglets is 28–35 days, whereas early weaning is around 21 days. As we know, in the large-scale swine industries, early weaning of piglets has been becoming more and more popular because the strategy can not only improve the sow turnover and the utilization of delivery rooms but also reduce the cost of production (25). It is also beneficial for avoiding the risk that comes from biosecurity. However, early weaning usually leads to a severe stress response in piglets, which affects their growth performance and health (26–28). To solve these problems, several feed additives have been used in recent years, especially natural products. Squalene (SQ) is a triterpenoid unsaturated hydrocarbon that plays a significant role in the synthesis of fat-soluble vitamins, hormones, and cholesterol in eukaryotes (29, 30). Studies have shown that oral squalene can improve the effect of cyclophosphamide on the growth performance of Wistar rats (31). Research by Chen showed that adding 1.0 g/kg of squalene to the diet of broilers can linearly improve ADG and F/G (32). Our experiment shows that adding 250 mg/kg of squalene to the basal diet can significantly improve the growth performance of early-weaned piglets, which is consistent with the results of previous studies on broilers (16, 32). Meanwhile, adding squalene to the diet of early-weaned piglets can significantly alleviate diarrhea (33). The main possible reason is that the all-trans 6 double bond structure of squalene makes it possible to penetrate the membrane structure of the organ and achieve uniform distribution, and therefore, some pathogenic bacteria cannot enter the animal body. Additionally, squalene has unsaturated double bonds, which can reduce inflammation (34), and it can be used to treat diarrhea. Squalene can not only repair the stress damage caused by early weaning but also improve the activity of antioxidant enzymes and relieve inflammation. Therefore, squalene can be regarded as a potential feed additive to improve the growth performance of weaned piglets.

The stresses of early weaning and environmental bacteria in piglets are significant factors that induce inflammation, which is often measured by the level of inflammatory factors in the serum. It is generally believed that TNF-α and IL-6 are pro-inflammatory factors that play important roles in promoting the occurrence of acute inflammation. In contrast, TGF-β, IL-10, and IFN-γ are anti-inflammatory factors. In this study, the addition of squalene to the diet significantly reduced the level of inflammatory factors in the serum of early-weaned piglets and increased the level of anti-inflammatory factors, which is consistent with the previous research (35, 36). After weaning, the nutrient source of piglets changes from milk to feed, the gut is not yet fully developed, and the immune function and defense capability are very weak, so different conditions of oxidative stress can easily occur (37). Antioxidant enzyme activity and MDA level in serum can reflect the degree of oxidative damage in early-weaned piglets (38). Studies have shown that adding squalene to the broiler diet can increase the activity of SOD and GSH-Px in the serum, thereby reducing the accumulation of MDA in the peripheral circulation (32). This study reported that SQ can significantly reduce the level of MDA in the serum of early-weaned piglets and significantly increase the level of antioxidant indicators, such as SOD, GSH-Px, CAT, and T-AOC, which is consistent with previous research (39). SQ can not only increase the antioxidant capacity in the blood but also increase it in the jejunum. Our data indicate that antioxidant indexes such as SOD, GSH-Px, and CAT were higher in the SQ group. In contrast, the level of MDA is lower in the treatment group. This means that adding squalene to the basal diet can improve the antioxidant performance in both the serum and the intestine of early-weaned piglets. Therefore, squalene can be regarded as a potential feed additive to alleviate weaning stress and reduce the occurrence of inflammatory responses in early-weaned piglets.

The gut barrier function consists of a tight junction and an adherens junction, which form a physical barrier to inhibit inflammatory infiltration and protect gut health (40). Once the barrier is broken, the intestinal tight junction (ZO-1, Occludin, and Claudin) of the epithelium cells is disrupted (41), and the intestinal villi, which are composed of goblet cells, are also reduced. Goblet cells are known to secrete mucus. The more goblet cells there are, the more mucus is secreted. The mucus can stick harmful bacteria to the surface and prevent them from entering the intestinal tract (42, 43). The relationship between SQ and the intestinal barrier has not yet been reported. However, dietary squalene supplementation can improve DSS-induced acute colitis (44). Many studies have shown that antioxidants can relieve colitis by improving the intestinal barrier of animals (45–47). Oral coffee acid, a kind of antioxidant (60 mg/kg body weight), could increase the expression of ZO-1 and Occludin against a high-fat diet (HFD)-induced hepatic steatosis and inflammation (48). In our study, the mRNA expressions of *ZO-1, Occludin*, and *Claudin* were all significantly increased in the treatment group compared to the CON group. Meanwhile, the mRNA expressions of *GLP-2* and *IGF-1* were both significantly increased in the SQ group. Studies have shown that GLP-2 could improve gut permeability to control inflammation in obese mice (49). In humans, a benefit of GLP-2 is that it repairs intestinal stem cells and Paneth cells during graft vs. host disease (50). Similarly, IGF-1 promoted colonic epithelial integrity and regeneration in colitis mice (51). It has also been reported that IGF-1 protects intestinal epithelial cells from oxidative-stress-induced apoptosis (52). Squalene is an antioxidant that can reduce post-weaning stress injury in piglets by increasing the IGF-1 content. Once the IGF-1 signaling pathway is activated, the intestinal mucosal barrier can improve (53). GLP-2 and IGF-1 are both related to promoting intestinal villi regeneration (54, 55). Therefore, the potential mechanism of squalene in improving the intestinal barrier of early-weaned piglets is to enhance the regeneration of small intestinal villi and increase the secretion of tight junction proteins.

The gut microbiota has many physiological roles, including not only metabolic-related disorders, such as obesity and diabetes (56, 57), but also diseases and conditions related to the nervous and reproductive systems (58). Based on all the results, we found that SQ supplementation could alter the microbial composition and inhibit inflammatory factors and oxidative stress. Intestinal microbes play a vital role in the process of gut development. A recent study has shown that gut microbiota can affect growth performance by adding food additives in animal models. The most commonly affected genera include *Blautia, Butyricicoccus, Gemmiger*, and *Holdemanella* (59). In our investigation, we also found that the abundance of *Gemmiger* increased significantly when we added SQ. Meanwhile, reducing harmful bacteria in the gut is also a means of improving animal growth performance. Research has shown that adding antioxidants can decrease the level of *Alloprevotella, Desulfovibrio*, and *Barnesiella* to relieve intestinal inflammation and reduce intestinal diseases and potentially pathogenic bacteria (60–62). In our present research, we found that *Alloprevotella, Desulfovibrio*, and *Barnesiella* were also the dominant microbiota in the CON group and that SQ supplementation could reduce its abundance. These deleterious effects were also reversed by SQ supplementation, indicating that SQ has the potential function of restoring the intestinal microbial community. Therefore, another potential mechanism by which SQ can improve the growth performance of early-weaned piglets is by increasing the beneficial bacteria and reducing the harmful bacteria in the intestine.

## Conclusion

In summary, we found that SQ benefited the growth performance of early-weaned piglets by improving the gut microbiota, intestinal barrier, and blood antioxidative stress. Therefore, SQ may be used as a dietary feed additive for piglets after weaning for improving growth performance. These improvements will increase the survival rate of early-weaned piglets and meet the growing demand for pork consumption.

## Data availability statement

The datasets presented in this study can be found in online repositories. The names of the repository/repositories and accession number(s) can be found in the article/Supplementary material.

## Ethics statement

The animal study was reviewed and approved by the Animal Care and Use Committee of Baicheng Normal University.

## Author contributions

YG and XM performed the experiments and analyzed the data. YG, XM, and YZ designed and supervised the study. YG, XM, YZ, YL, and DX wrote the manuscript. YG revised the manuscript. All authors edited the manuscript and approved the final manuscript.

## Funding

This research was supported by Baicheng Normal University Doctoral Research Project (012006) and Jilin Province Science and Technology Department Project (JJKH20190017KJ).

## Conflict of interest

Author DX was employed by Muyuan Joint Stock Company. The remaining authors declare that the research was conducted in the absence of any commercial or financial relationships that could be construed as a potential conflict of interest.

## Publisher's note

All claims expressed in this article are solely those of the authors and do not necessarily represent those of their affiliated organizations, or those of the publisher, the editors and the reviewers. Any product that may be evaluated in this article, or claim that may be made by its manufacturer, is not guaranteed or endorsed by the publisher.

## Supplementary material

Supplementary Table 1 Composition and nutrient levels of basal diets (air-dry basis).

Supplementary Table 2 Information for primary antibodies.

Supplementary Table 3 Raw data of microbiota in genus level top 20.

Supplementary Table 4 Raw data of microbiota in genus level including others.

Supplementary Table 5 Spearman's correlation coefficient table.
