# Supplementary material for: Dietary supplementation of squalene increases the growth performance of early-weaned piglets by improving gut microbiota, intestinal barrier, and blood antioxidant capacity
Source: Front Vet Sci. 2022 Nov 3;9:995548. doi: 10.3389/fvets.2022.995548 (PMC9669083; doi:10.3389/fvets.2022.995548)
Supplement: Supplementary Table 1 — Composition and nutrient levels of basal diets (air-dry basis). [file Table_1.docx]

Table 1. Composition and nutrient levels of basal diets (air-dry basis)

| Composition | Content（%） | Nutrition level ^2)^ | |
| --- | --- | --- | --- |
| Corn | 67.60 | DE（MJ/kg） | 13.80 |
| Fish meal | 8.00 | CP（%） | 20.01 |
| Whey | 6.00 | Ca（%） | 0.80 |
| Soy | 15.50 | TP（%） | 0.60 |
| Lysine hydrochloride | 0.15 | AP（%） | 0.40 |
| CaHP0_4_ | 0.40 | Lys（%） | 1.35 |
| Limestone | 0.95 | Met（%） | 0.40 |
| NaCl | 0.22 | Thr（%） | 0.85 |
| Choline chloride | 0.18 | Trp（%） | 0.23 |
| Permix^1)^ | 1.00 |  |  |
| Total | 100.00 |  |  |

^1)^ Supplied the following per kilogram of completed diet: Fe 100 mg, Cu 80 mg, Zn 100 mg, Mn 60 mg, I 0.3 mg, Se 0.25 mg, Vitamin A 10000 IU, Vitamin D3 1400 IU, Vitamin E 40 mg, Vitamin K3 2.50 mg, thiamin 1.50 mg, riboflavin 5.0 mg, pyridoxine 4.0 mg, vitamin B12 0.02 mg, niacin 25 mg, pantothenic calcium 12 mg, folacin 0.60 mg, biotin 0.10 mg

^2)^ CP, Ca and TP were measured, and the rest were calculated values.
