# Supplementary material for: Dietary supplementation of squalene increases the growth performance of early-weaned piglets by improving gut microbiota, intestinal barrier, and blood antioxidant capacity
Source: Front Vet Sci. 2022 Nov 3;9:995548. doi: 10.3389/fvets.2022.995548 (PMC9669083; doi:10.3389/fvets.2022.995548)
Supplement: Supplementary Table 2 — Information for primary antibodies. [file Table_2.docx]

**Table S2 Primer Sequence**

| Gene name | Primer sequence (5’–3’) | Size（bp） |
| --- | --- | --- |
| *GAPDH* | F: 5’-TGCCATCAATGACCCCTTC-3’ | 182 |
|  | R: 5’-CACCAGCATCACCCCACTT-3’ |  |
| *GLP-2* | F: 5’-ACTCACAGGGCACGTTTACCA -3 | 149 |
|  | R: 5’-AGGTCCCTTCAGCATGTCTCT -3’ |  |
| *IGF-1* | F: 5’-CTGAGGAGGCTGGAGATGTACT -3’ | 137 |
|  | R: 5’-CCTGAACTCCCTCTACTTGTGTTC-3’ |  |
| *Claudin* | F: 5’- AGGACTACGTATGAGGGGGC -3’ | 99 |
|  | R: 5’- GACTGGTCTCGGATGCAAGG -3’ |  |
| *Occludin* | F: 5’- CAGGTGCACCCTCCAGATTG -3’ | 74 |
|  | R: 5’- AGCGGGTCACCTGATCTTCA -3’ |  |
| *ZO-1* | F: 5’- ACAGTGCCCAGAGACCAAGA -3’ | 87 |
|  | R: 5’- CATTTCCTCGGGGTAGGGGT -3’ |  |
